# *MLIP* and Its Potential Influence on Key Oncogenic Pathways

**DOI:** 10.3390/cells13131109

**Published:** 2024-06-26

**Authors:** Mahmoud N. Hamwi, Engy Elsayed, Hanan Dabash, Amani Abuawad, Noor A. Aweer, Faissal Al Zeir, Shona Pedersen, Layla Al-Mansoori, Patrick G. Burgon

**Affiliations:** 1 College of Medicine, Qatar University, Doha P.O. Box 0974, Qatar; mh1805711@student.qu.edu.qa (M.N.H.); ee1904424@student.qu.edu.qa (E.E.); na2003836@student.qu.edu.qa (N.A.A.); fa2105843@student.qu.edu.qa (F.A.Z.); spedersen@qu.edu.qa (S.P.); 2 Department of Chemistry and Earth Sciences, College of Arts and Sciences, Qatar University, Doha P.O. Box 2713, Qatar; hd1705242@student.qu.edu.qa (H.D.); aa1801473@student.qu.edu.qa (A.A.); 3 Biomedical Research Centre, Qatar University, Doha P.O. Box 2713, Qatar

**Keywords:** *MLIP*, cancer, PI3 kinase, *Akt*, *mTOR*, tumorigenesis

## Abstract

Muscle-enriched A-type lamin-interacting protein (*MLIP*) is an emerging protein involved in cellular homeostasis and stress adaptation. Eukaryotic cells regulate various cellular processes, including metabolism, DNA repair, and cell cycle progression, to maintain cellular homeostasis. Disruptions in this homeostasis can lead to diseases such as cancer, characterized by uncontrolled cell growth and division. This review aims to explore for the first time the unique role *MLIP* may play in cancer development and progression, given its interactions with the *PI3K/Akt/mTOR* pathway, *p53*, *MAPK9*, and *FOXO* transcription factors, all critical regulators of cellular homeostasis and tumor suppression. We discuss the current understanding of *MLIP*’s involvement in pro-survival pathways and its potential implications in cancer cells’ metabolic remodeling and dysregulated homeostasis. Additionally, we examine the potential of *MLIP* as a novel therapeutic target for cancer treatment. This review aims to shed light on *MLIP*’s potential impact on cancer biology and contribute to developing innovative therapeutic strategies.

## 1. Introduction

Eukaryotic cells maintain cellular homeostasis through an extensive array of sensory mechanisms to respond and adapt to both intrinsic and extrinsic stimuli and insults. This involves the regulation of various cellular processes, including metabolism, DNA repair, and cell cycle progression. Disruptions to cellular homeostasis can lead to the development of various diseases, including cancer. Cancer is a devasting disease with metabolic remodeling and dysregulated homeostasis as distinctive features [1] and is characterized by uncontrolled cell growth and division, leading to the formation of tumors [2]. This process is the result of a disruption in the delicate balance between cell proliferation and cell death [3], which is normally maintained by cellular homeostasis. Several signaling molecules and pathways have been identified as pro-oncogenic and have therefore been targeted for the therapeutic treatment of cancer [4].

The integrated actions of the tumor suppressors *p53 (p53)*, *FOXO*, and the *PI3K/Akt/mTOR* pathway enable cells to respond to a wide range of stresses, including oxidative stress, nutrient deprivation, and DNA damage. This integrated stress response is crucial for maintaining cellular and organismal homeostasis. In cancer cells, however, mutations or changes in the genes encoding proteins in this pathway can cause hyperactivation, resulting in uncontrolled cell growth and resistance to apoptosis (programmed cell death). For example, the phosphoinositide 3-kinase/protein kinase B (*PI3K/Akt*)/mammalian target of rapamycin (*mTOR*) pathway is tightly regulated in normal cells, ensuring a balance between cell growth and death. However, *p53* and forkhead box O family (*FOXO*), downstream of the *PI3K/Akt/mTOR* pathway, respectively, are critical integrators of genomic and metabolic stresses [5,6,7,8]. Both *p53* and *FOXO* are stress-activated transcription factors that promote an adaptive pro-survival response to insult [9,10,11]. Specifically, *p53* stimulates DNA repair in response to DNA damage [12,13] and *FOXO* regulates metabolic remodeling to maintain metabolic homeostasis [14,15,16].

Cancer metabolism modifies cellular metabolic pathways to facilitate rapid cell growth and proliferation [17,18,19]. Cancer cells undergo metabolic reprogramming to meet the high energy and biosynthetic demands associated with their fast growth. This reprogramming promotes glycolysis at the expense of oxidative phosphorylation, a phenomenon known as the “Warburg effect”, which occurs even in the presence of oxygen. The “Warburg effect” is the preference of cancer cells to utilize aerobic glycolysis instead of oxidative phosphorylation for energy generation [20,21]. Tumor cells derive advantages from this alteration in metabolism. Initially, glycolysis generates energy at a quicker rate compared to oxidative phosphorylation, although producing a lower amount of ATP. Additionally, glycolysis generates intermediary compounds that are crucial for the synthesis of nucleotides, amino acids, and lipids, all of which play vital roles in the growth and multiplication of cells. Furthermore, cancer cells reduce the production of reactive oxygen species (ROS) and mitigate oxidative stress and apoptosis by minimizing mitochondrial respiration. *P53*, the *PI3K/Akt*/*mTOR* pathway and the *FOXO* transcription factor have all been implicated in the metabolic remodeling that cancer cells undergo during tumorigenesis [20,22,23,24,25].

Muscle enriched A-type lamin-interacting protein (*MLIP*) is a novel protein of unknown structure and function that is required for the proper cardiac and skeletal muscle adaptation to stress [26,27,28,29,30,31]. *MLIP* is a crucial mediator of cardiac adaptation through its interaction with the *Akt*/*mTOR* pro-survival pathway [28], *FOXO*1 [31] and *p53* [28]. A detailed comparative pathway analysis based on global gene expression differences between normal and *MLIP*-deficient hearts has now revealed *MLIP* as a modulator of both *p53* and *FOXO* activity. Given *MLIP*’s interactions with the *PI3K/Akt* pathway, *p53*, and *FOXO* [28,32], this review explores the roles *MLIP* may play in tumor formation and progression and the potential of *MLIP* as a new therapeutic target. The focus of this review is to explore the relationship of *MLIP* with well characterized factors that are associated with the development and progression of cancer.

## 2. *MLIP* Discovery and Cellular Role

The discovery of muscle-enriched A-type lamin-interacting protein (*MLIP*) [27] marks a significant advancement in understanding the molecular intricacies associated with laminopathies—degenerative disorders tied to mutations in the *LMNA* gene. This discovery sheds light on *MLIP*’s evolution, as it is uniquely found in amniotes [27], suggesting its emergence as an evolutionary novelty in these organisms. *MLIP* is characterized by alternatively spliced variants [27,29], showing a broad expression spectrum with a predominance in muscular tissues. Its interaction with lamin A/C and co-localization with promyelocytic leukemia (PML) bodies [27] suggest a vital role in cellular organization and differentiation, hinting at its contribution to the mesenchymal phenotypes observed in laminopathies.

Parallel to the original discovery of *MLIP*, the identification of cardiac Isl1-interacting protein (*CIP*, an alias of *MLIP*) [33] provided insights into cardiac hypertrophy’s regulatory mechanisms [34], underscoring its repressive role in cardiomyocyte hypertrophy and downregulation in hypertrophic conditions. *CIP*’s interaction with Isl1, essential for cardiac progenitor specification [34], emphasizes its regulatory capacity in cardiac development and hypertrophy. Further studies elucidated *MLIP*’s essential roles in cardiac and skeletal muscle function. For instance, *MLIP* deletion or overexpression significantly impacted the *Akt*/*mTOR* pathways [28], highlighting its potential as a cardiac stress sensor. This insight and genome-wide association studies linking *MLIP* to the cardiac stress response [28] underscores its therapeutic potential in cardiac diseases.

The exploration into *MLIP*’s function revealed its interaction with chromatin [26], suggesting a role in regulating muscle-specific gene networks and highlighting its importance in muscle differentiation and maintenance. This chromatin association and its ability to influence myoblast differentiation [26] positions *MLIP* as a critical factor in muscle physiology [30]. Moreover, studies revealed *MLIP*’s potential link to cancer through its involvement in gene networks related to cellular stress responses, including those mediated by *p53* [28] and *FOXO* [31]. Such connections suggest a broader role for *MLIP* in cellular homeostasis and disease, including potential implications in cancer biology.

## 3. *MLIP* Expression in Cancer

Limited research has focused on elucidating the role of *MLIP* in the initiation and/or progression of cancer. Our investigation identified two primary types of cancers where *MLIP*’s role was emphasized: breast cancer and esophageal cancer (Table 1).

Breast cancer and esophageal cancer represent significant global health challenges, with the former being one of the most prevalent cancers among women and the latter noted for its particularly low survival rates [38,39]. The genetic underpinnings of these cancers are complex, and though substantial progress has been made in identifying key genetic risk factors, a significant proportion of the genetic risk remains unexplained. Recent research has begun to shed light on this gap, with a particular focus on the role of copy number variants (CNVs) and differentially expressed genes. One gene that has emerged as a potential key player in both breast and esophageal cancer is the *MLIP* gene.

### Expression of MLIP in Different Types of Cancer

Breast cancer stands as one of the prevalent malignancies affecting women, with around one million new cases and over 400,000 reported deaths annually worldwide. In the year 2023, an estimated 297,790 women and 2800 men were projected to be diagnosed with breast cancer [40]. While single nucleotide polymorphisms and mutations contribute to approximately 49% of the genetic risk associated with breast cancer [41,42], Kumaran and colleagues (2017) sought to uncover the remaining 51% by identifying germline copy number variants (CNVs) linked to breast cancer [39]. Whole genome CNV genotyping was performed on 422 cases and 348 controls. Two hundred CNVs were identified to be associated with breast cancer of which 21 CNV regions overlapped with 22 genes. *MLIP* was identified as one of six genes associated with the breast cancer risk and recurrence-free survival [39]. Specifically, Kumaran and colleagues reported that a loss in *MLIP* CNVs was associated with significant reduction in the breast cancer risk and recurrence-free survival, with a reported hazard ratio of 0.62 [0.4–0.94] [39].

Triple-negative breast cancer (TNBC) is an aggressive subtype of breast cancer that is defined by the absence of estrogen receptor, progesterone receptor, and human epidermal growth factor receptor 2 expression. These receptors are commonly used as targets for breast cancer treatment, and the absence of these receptors in TNBC makes it more difficult to treat. Zhang and colleagues performed RNA-seq on 30 TNBC patient tumors, 15 of which had lymph node metastasis while the other 15 showed no lymph node metastasis [32]. Differential gene expression analysis was performed in order to determine the key genes involved in the progression and oncogenesis of TNBC [32]. The analysis revealed 2953 genes with differential expression in breast tumors compared to normal control tissues and 975 genes with differential expression between 15 patients with lymph node metastasis and 15 patients without. A subset of 117 genes exhibited differential expression in both sets among those with and without lymph node metastasis in triple-negative breast cancer (TNBC), implying their involvement in TNBC oncogenesis and progression. Among the 117 genes of interest, *MLIP* was found to be upregulated in TNBC and exhibited a negative association with the cytotoxicity of CD8+ T cells [32].

Esophageal cancer is one of the most common malignancies, ranking seventh in global morbidity and sixth in cancer-related mortality. The 5-year overall survival rate is only about 15–20%, although progress has been made in diagnosis and treatment [38]. To further define prognostic mRNAs of esophageal cancer, functional enrichment analyses of lncRNA, mRNA, and miRNA in 81 tumors and 11 normal control tissues was performed. *MLIP* was identified as one of seven risk RNAs for esophageal cancer with a hazard ratio of 1.67 (1.22–2.29, *p* < 0.001) [38].

Finally, according to the recently found role of *MLIP* in cancer (Table 1), it has been suggested as a potential biomarker for triple-negative breast cancer and esophageal cancer. However, more research is needed to fully understand *MLIP*’s role in these cancers and its potential as a therapeutic target or diagnostic tool.

## 4. Molecular Relationship of *MLIP* with Pro-Survival/Oncogenic Pathways and Tumor Suppressors

**Figure 1:**
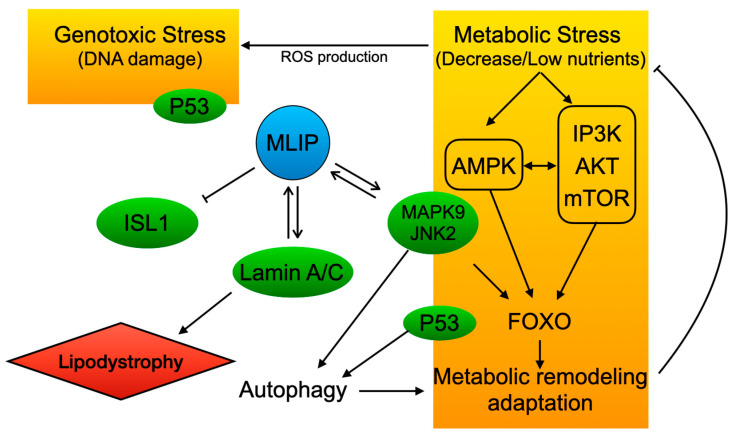
Schematic representation of *MLIP*-associated stress response pathways in cellular homeostasis and disease. The central role of *MLIP* (muscle-enriched A-type lamin-interacting protein) in coordinating cellular responses to genotoxic and metabolic stress is shown, highlighting its potential implications in cancer.

The intricate network of cellular signaling pathways that govern cell growth, proliferation, survival, and metabolism is often dysregulated in various cancer types, contributing to tumorigenesis and disease progression. Central to this network are the *PI3K/Akt*/*mTOR*, *FOXO*, *AMPK*, *p53*, and lamin A/C pathways, each playing critical roles in maintaining cellular homeostasis and responding to stress signals. Recently, *MLIP* has emerged as a key interactor/regulator of the *PI3K/Akt*/*mTOR*, *FOXO*, *AMPK*, *p53*, and lamin A/C pathways (Figure 1), influencing a variety of cellular processes and potentially playing roles in both cancer pathogenesis and cardiac disorders.

### 4.1. MLIP: AMPK Function and Dysfunction in Cancer

The study by Cattin et al. in 2015 sheds light on the molecular mechanisms underlying the reduced glucose uptake observed in *MLIP*-deficient cardiac tissues [28]. In *MLIP*-deficient hearts, adenosine monophosphate-activated protein kinase (*AMPK*) was reported to undergo dephosphorylation at *AMPK* alpha-Thr-172, a crucial step leading to the deactivation of the *AMPK* complex and subsequently resulting in decreased glucose uptake compared to normal cardiac tissues [28]. Remarkably, this deactivation of *AMPK* occurred despite similar activity of liver kinase B1 (LKB1), the kinase responsible for *AMPK* activation [43], indicating an LKB1-independent inactivation of *AMPK* in *MLIP*-deficient hearts.

*AMPK* serves as a pivotal enzyme governing the cellular energy balance. Its primary function involves detecting shifts in the cellular energy status, particularly reductions in ATP, and initiating processes that generate ATP while concurrently inhibiting ATP-consuming processes. *AMPK* functions as a heterotrimeric complex, comprising catalytic α subunits and regulatory β and γ subunits. The γ subunit accommodates binding sites for AMP and ATP, enabling *AMPK* to sense alterations in the AMP/ATP ratio and self-activate during energy depletion. *AMPK* activation triggers diverse downstream effects, including heightened glucose uptake, fatty acid oxidation, and mitochondrial biogenesis, along with diminished protein synthesis, lipogenesis, and gluconeogenesis. *AMPK* also influences autophagy, cell growth, proliferation, and inflammation [44,45]. In response to stressors causing ATP depletion, such as hypoxia and glucose deprivation, *AMPK* activity is heightened [44,45]. Additionally, stimulating *AMPK* in skeletal muscle enhances glucose uptake and fatty acid oxidation while reducing lipid accumulation and inflammation [46]. These findings, combined with other research, collectively underscore the crucial role of *AMPK* in governing energy metabolism and cellular function.

**Figure 2:**
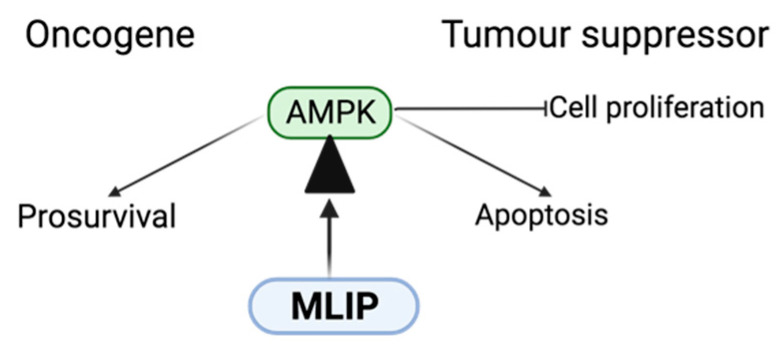
Regulatory interactions between *MLIP* and *AMPK* in cancer metabolism. This schematic illustrates the bidirectional regulatory relationship between muscle-enriched A-type lamin-interacting protein (*MLIP*) and AMP-activated protein kinase (*AMPK*). *MLIP* is depicted as acting on the fulcrum of *AMPK* action as an oncogene or tumor suppressor.

The precise function of *AMPK* in cancer cells is complicated and relies on the specific context of *AMPK* activation. In certain instances, *AMPK* activation can serve as a tumor suppressor by restraining cell growth, curbing proliferation, and encouraging apoptosis. However, in other scenarios, *AMPK* activation might support the survival of tumor cells by facilitating metabolic adaptation to the unique conditions of the tumor microenvironment. Hence, targeting *AMPK* activation could be a problematic or promising approach for cancer treatment (Figure 2) [47]. Additionally, research indicates that combining *AMPK* activation with other anticancer therapies like chemotherapy or radiation has the potential to augment their effectiveness [47,48].

Interactions between *MLIP* and *AMPK* may hold implications for cancer biology. *AMPK*, recognized as a metabolic tumor suppressor, hampers cell growth and proliferation during low energy conditions, thereby impeding the uncontrolled cell growth characteristic of cancer [49]. Consequently, the observed reduction in *AMPK* activation in the absence of *MLIP* might potentially elevate the risk of unregulated cell growth and proliferation, contributing to oncogenesis. Furthermore, the decline in *AMPK* levels in *MLIP*-deficient cardiac tissues led to the heightened activation of the *Akt*/*mTOR* pathway [28,50]. This pathway significantly influences cell growth, proliferation, and survival, and its dysregulation is commonly observed in various types of cancers. These findings suggest that *MLIP* could potentially modulate these crucial pathways, thereby influencing cancer biology [28,49,50]. However, it is crucial to acknowledge that these observations were made specifically in cardiac tissue, and it remains uncertain whether similar mechanisms would apply to other tissues or cancer cells. Additional research is required to directly investigate the involvement of *MLIP* in cancer biology.

### 4.2. MLIP and the PI3K/Akt/mTOR Pathway

**Figure 3:**
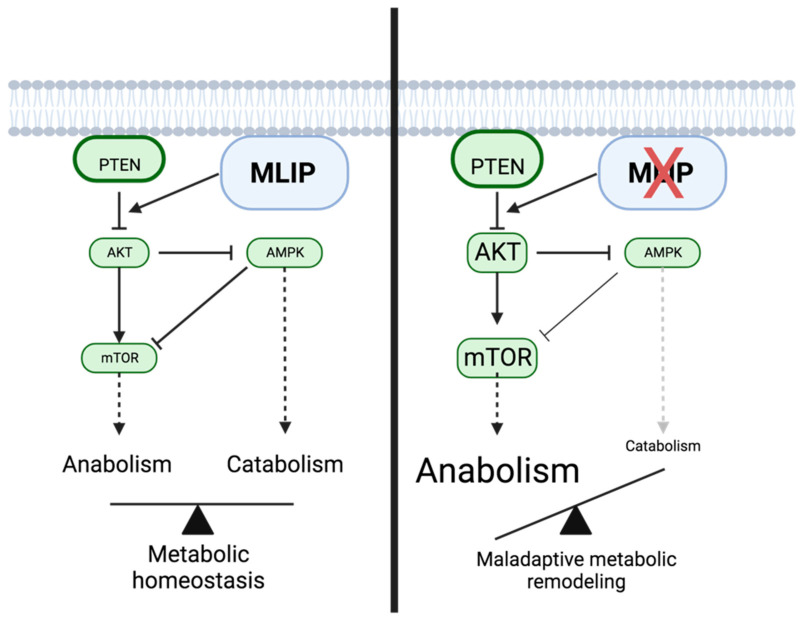
Comparative schematic of *MLIP* signaling pathways in physiological and pathological states. A comparative overview of the intracellular signaling pathways involving muscle-enriched A-type lamin-interacting protein (*MLIP*) in two states: physiological (**left panel**) and pathological (**right panel**). In the physiological state, *MLIP* negatively regulates *AKT*, leading to reduced *mTOR* activity and an increase in *AMPK* activity, depicted by solid black lines. These interactions suggest a role for *MLIP* in maintaining the cellular energy balance and potentially inhibiting cancer cell growth. In the pathological state (**right panel**), the loss of *MLIP* leads to increased *AKT*-*mTOR* activity, potentially promoting cell growth and proliferation. The altered signaling dynamics of *MLIP* in the pathological state underscore its potential as a regulatory switch in cancer metabolism and a target for therapeutic intervention. Figure created with BioRender.com. Reprinted with permission from Ref. [28]. Copyright 2015 Creative Commons CC-BY license.

The documented association between *MLIP* and the *PI3K/AKT*/*mTOR* signaling pathway is evident in research findings that highlight *MLIP*’s direct impact on this pathway [28]. Specifically, the absence of *MLIP* leads to the selective hyperactivation of the *Akt*/*mTOR* signaling pathway in cardiac cells (Figure 3) [28]. Conversely, *MLIP* overexpression results in the inhibition of this pathway. The study demonstrates that the hyperactivation of *Akt*/*mTOR* occurs in cardiac cells when *MLIP* is absent [28]. These results suggest that a deficiency in *MLIP* may potentially contribute to an accelerated aging phenomenon within cardiac cells, heightening susceptibility to tumor development.

**Figure 4:**
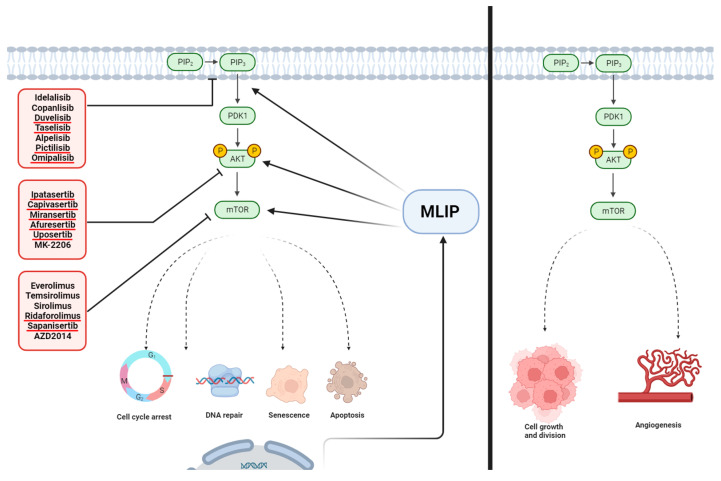
Influence of *MLIP* on key signaling pathways and therapeutic interventions in cancer. This figure delineates the role of muscle-enriched A-type lamin-interacting protein (*MLIP*) in modulating critical signaling cascades involved in cancer pathophysiology. In the left panel, the *MLIP* interaction with the *PI3K/AKT*/*mTOR* pathway is illustrated, depicting potential points of therapeutic intervention with the listed drugs. The dashed lines represent indirect effects on downstream processes such as cell cycle arrest, DNA repair, senescence, and apoptosis, which are key cellular responses in cancer therapy. In the right panel, the absence of *MLIP*’s regulatory influence is shown to result in enhanced cell growth and division, as well as angiogenesis, contributing to tumor progression. The drugs targeting these pathways are grouped according to their mechanism of action, highlighting the potential for *MLIP* to serve as a pivotal point for therapeutic targeting in cancer treatment. Figure created with BioRender.com.

The regulatory link between *MLIP* and the *PI3K/AKT*/*mTOR* pathway is underscored by research indicating *MLIP*’s direct influence on this critical signaling pathway [28]. The absence of *MLIP* leads to the selective hyperactivation of the *Akt*/*mTOR* pathway in cardiac cells, as evidenced by the significant increase in pathway activity in *MLIP*-deficient cells. Conversely, *MLIP* overexpression results in the inhibition of this pathway, highlighting *MLIP*’s role as a negative regulator [28,31]. This hyperactivation of the *Akt*/*mTOR* pathway in the absence of *MLIP* suggests that *MLIP* deficiency may accelerate aging in cells and may heighten their susceptibility to tumor development, potentially implicating *MLIP* in cancer biology. The *PI3K/Akt*/*mTOR* pathway is crucial for regulating cellular processes such as growth, proliferation, survival, and metabolism, and its dysregulation is frequently observed in various cancers. Activation of this pathway in cancer cells can occur through multiple mechanisms, including genetic mutations, activation of upstream growth factor receptors, and loss of negative regulators. This activation promotes increased cell proliferation, survival, and resistance to cell death, contributing to tumor growth and progression (Figure 4) [51,52,53,54]. Thus, *MLIP* deficiency could facilitate these oncogenic processes, suggesting that *MLIP* might be a novel target for cancer therapy. Targeting the *PI3K/Akt*/*mTOR* pathway (Table 2) has become a promising cancer treatment strategy [53,54], with several drugs in development and testing showing potential in preclinical and clinical studies. However, these therapies must balance effectiveness with minimizing toxicity, necessitating ongoing research to improve their selectivity and efficacy [51,53,54]. Understanding *MLIP*’s regulatory role may offer new insights into cancer treatment strategies, potentially leading to more effective interventions.

### 4.3. Role of MLIP in FOXO1 Signaling

*FOXO* genes are a subgroup of the forkhead family of transcription factors that play a critical role in regulating various cellular processes, including cell cycle control, DNA repair, apoptosis, and the oxidative stress response [86,87,88,89]. Notably, the deletion of *MLIP* has also been linked to the downregulation of the *FOXO*1 pathway [28,31]. This suggests that the transcription factor *FOXO*-1 operates as a downstream signal of *MLIP*.

Dysregulation of *FOXO* gene expression or activity has been reported to be associated with the development and progression of cancer [90]. There are four members of the *FOXO* family in mammals: *FOXO*1, *FOXO*3, *FOXO*4, and *FOXO*6. Among these, *FOXO*1 and *FOXO*3 are the most well-studied in the context of cancer (Table 3). In normal cells, *FOXO*1 and *FOXO*3 are often activated in response to cellular stress, leading to the expression of target genes that promote cell cycle arrest, DNA repair, and apoptosis. This helps to prevent the development of cancer by eliminating cells with damaged DNA [91]. However, in cancer cells, the activity of *FOXO*1 and *FOXO*3 is often dysregulated [92,93,94]. In tumors, *FOXO* expression or activity is often suppressed to promote cell proliferation and survival, or alternatively, *FOXO* may be activated to promote cell migration and invasion [92,93,94].

*FOXO*1 has been found to play a role in the regulation of estrogen receptor (ER) signaling. In breast cancer, the loss of *FOXO*1 activity is associated with resistance to endocrine therapy, while overexpression of *FOXO*1 has been shown to sensitize breast cancer cells to endocrine therapy [35]. Likewise, in prostate cancer, *FOXO*3 has been identified as a parti*CIP*ant in the control of androgen receptor signaling [94]. Reduced *FOXO*3 activity has been linked to resistance to androgen deprivation therapy, whereas increased *FOXO*3 expression has demonstrated the ability to enhance the sensitivity of prostate cancer cells to this therapy.

Although the precise mechanism through which *MLIP* increases *FOXO*-1 expression remains unknown, *FOXO*-1 is acknowledged for its involvement in cell cycle arrest, apoptosis, and tumor suppression, implying a potential role for *MLIP* in cancer pathogenesis. The activation of *FOXO*1 prompts the transcription of the cyclin-dependent kinase inhibitor p27^KIP1^ while suppressing the transcription of cyclin D1 and D2. Both effects result in cell cycle arrest at G1 phase. The loss of one allele of *FOXO* may render cells susceptible to dysregulated cell cycle events, triggering tumor formation. Activation of *MLIP* may mitigate the impact of *FOXO* haploinsufficiency on tumorigenesis.

### 4.4. MLIP and P53

*MLIP*-deficient hearts were found to have increased activation of *p53* [28], indicating that *MLIP*-deficient hearts may be experiencing genotoxic and/or metabolic stress. The *p53* gene functions as a crucial tumor suppressor, actively preventing cancer development by regulating various cellular processes, including DNA repair, cell cycle arrest, apoptosis, and senescence [7,8,37,95,96,97]. In response to DNA damage, *p53* is activated, enabling it to pause the cell cycle for DNA repair or initiate apoptosis to eliminate damaged cells. In cancer, the *p53* gene is frequently mutated or deleted, resulting in the loss of its tumor suppressor function [12,98]. Mutations in *p53* represent one of the most prevalent genetic alterations in cancer, with up to 50% of all human cancers exhibiting *p53* mutations [99,100]. The functional loss of *p53* contributes to cancer development and progression by allowing the proliferation of damaged cells, facilitating the accumulation of additional genetic changes that can lead to cancer formation.

Beyond its role in the DNA damage response, *p53* also parti*CIP*ates in the regulation of cellular metabolism [101,102,103]. *P53* has been demonstrated to influence the expression of genes involved in glycolysis, oxidative phosphorylation [104,105], and fatty acid metabolism [101,106]. *P53* loss or mutation can contribute to the metabolic rewiring commonly observed in cancer cells [107]. However, the activation of *p53* is triggered by other genes and is crucial for its role as a tumor suppressor [8]. This implies a potential alternative function of *MLIP*, wherein it may promote tumor formation by inhibiting *p53*, a critical tumor suppressor gene. Alternatively, *MLIP* inhibition might impact *p53* function by influencing other genes associated with *p53* activation.

Based on the findings of increased activation of *p53* in *MLIP*-deficient hearts, one can speculate that *MLIP* may play a role in modulating *p53* function in cancer. This potential role could involve *MLIP* influencing *p53* activation and its associated pathways, either through direct interaction or by impacting other genes involved in *p53* regulation. Understanding this relationship may provide novel insights into how *MLIP* contributes to cancer development and progression, particularly through its interaction with the crucial tumor suppressor gene *p53*.

### 4.5. MLIP and MAPK9 (Jak2)

The global mapping of the human binary protein interactome revealed and confirmed the interaction between *MLIP* and *MAPK9* [108]. *MAPK9*, also known as *JNK2*, interacts intricately with several key signaling pathways, significantly impacting cellular processes and cancer biology [109,110]. One of its crucial interactions is with the *PI3K/Akt*/*mTOR* pathway, a major regulator of cell growth, proliferation, and survival [3,111,112]. *MAPK9* modulates this pathway through phosphorylation events, often inhibiting *PI3K/Akt* activity under stress conditions and thus promoting apoptosis over cell survival [111]. This interaction can form a negative feedback loop that counterbalances the pro-survival signals from *PI3K/Akt*/*mTOR*, maintaining cellular equilibrium.

In relation to the tumor suppressor *p53*, *MAPK9* plays a pivotal role in enhancing *p53* activity through phosphorylation [113,114]. This phosphorylation stabilizes *p53*, particularly in response to cellular stress and DNA damage, leading to the activation of *p53*-dependent apoptotic genes [113,115]. Through this mechanism, *MAPK9* contributes to the elimination of cells with damaged DNA, acting as a barrier against malignant transformation.

*MAPK9* also interacts with *FOXO* transcription factors, which are key regulators of apoptosis, cell cycle arrest, and oxidative stress resistance. *MAPK9* phosphorylates *FOXO* proteins, promoting their nuclear translocation and subsequent activation of stress response genes [111]. This regulation by *MAPK9* is crucial for cellular responses to oxidative damage, helping to maintain cellular integrity and prevent uncontrolled proliferation. Overall, *MAPK9*′s interactions with *MLIP*, the *PI3K/Akt*/*mTOR* pathway, *p53*, and *FOXO* transcription factors create a complex network (Figure 1) that governs cell survival, apoptosis, and stress responses. These molecular relationships underscore how *MLIP* may have a multifaceted role in maintaining cellular homeostasis and its possible role in cancer progression.

## 5. Conclusions and *MLIP* as a Potential Therapeutic Target

*MLIP* is an emerging factor implicated in the regulation of key signaling pathways that govern cell growth, proliferation, survival, and metabolism, which are often dysregulated in cancer. Through its interactions with the *PI3K/Akt*/*mTOR* pathway, *MLIP* appears to exert an inhibitory effect [28]. Overexpression of *MLIP* leads to the downregulation of this pathway, while its loss results in the pathway’s overactivation [28,31,34,116]. This implies that *MLIP* might act as a suppressor of cell growth and proliferation, two key processes that are often hyperactivated in cancer. Therefore, therapies aimed at enhancing *MLIP* expression or its regulatory effect on the *PI3K/Akt*/*mTOR* pathway might be beneficial for inhibiting cancer progression.

Moreover, *MLIP* appears to be involved in the regulation of *FOXO*1 signaling [31], a pathway that plays critical roles in cell cycle control, apoptosis, and DNA repair—processes that are crucial for maintaining genomic integrity and preventing tumorigenesis. Dysregulation of *FOXO*1 signaling is often associated with cancer progression. Given that the deletion of *MLIP* leads to a downregulation of the *FOXO*1 pathway, and overexpression of *MLIP* is likely to have the opposite effect, therapeutics aimed at enhancing *MLIP* function or expression could potentially restore the normal function of *FOXO*1 signaling, thereby inhibiting cancer development and progression.

Additionally, *MLIP*’s interactions with *p53* [28], a well-known tumor suppressor gene, further underscore its potential as a therapeutic target. Given that *MLIP*-deficient cardiomyocytes showed an increased expression of *p53*, it is plausible to hypothesize that *MLIP* could play a role in the regulation of *p53*, and by extension, cell cycle control and apoptosis.

However, it is essential to remember that the exact mechanisms of *MLIP* in these signaling pathways are not fully understood, and further research is necessary to establish *MLIP* as a therapeutic target. Furthermore, it is crucial to understand the potential off-target effects and safety profile of any *MLIP*-targeting therapies due to *MLIP*’s role in non-cancerous cells and processes, especially within the context of cardiac tissue where *MLIP* deficiency increase the susceptibility to developing heart failure [28,31] In summary, the modulation of *MLIP*’s function or its interactions with key signaling pathways represents a promising approach for the development of novel cancer therapeutics.

## Figures and Tables

**Table 1:** Association of *MLIP* with various cancer types and clinical outcomes.

Cancer	Status	Reference
Breast cancer	1 out of 6 genes associated with breast cancer risk and recurrence-free survival	[35]
Triple-negative breast cancer	Upregulated in and associated with patient survival in triple-negative breast cancer	[36]
Esophageal cancer	One of seven risk RNAs for esophageal cancer	[37]

**Table 2:** Overview of targeted therapies acting on the *PI3K/AKT*/*mTOR* pathway in cancer.

Drug	Molecular Target	Status	Reference
Idelalisib (Zydelig)	PI3K	relapsed chronic lymphocytic leukemia (CLL), follicular lymphoma, and small lymphocytic lymphoma (SLL)	[55]
Copanlisib (Aliqopa)	PI3K	relapsed follicular lymphoma	[56]
Duvelisib (Copiktra)	PI3K	relapsed or refractory CLL, SLL, and follicular lymphoma	[57]
Taselisib (GDC-0032)	PI3K	investigational drug in clinical trials for various types of cancer, including breast cancer and lung cancer	[58]
Alpelisib (Piqray)	PI3K	HR-positive, HER2-negative, PIK3CA-mutated advanced or metastatic breast cancer	[59,60]
Pictilisib (GDC-0941)	PI3K	investigational drug in clinical trials for various types of cancer, including breast cancer and non-small cell lung cancer	[61]
Omipalisib (GSK2126458)	PI3K	investigational drug in clinical trials for various types of cancer, including melanoma and pancreatic cancer	[62]
Ipatasertib (GDC-0068)	*AKT*	investigational drug in clinical trials for various types of cancer, including breast cancer, prostate cancer, and ovarian cancer	[63,64]
Capivaserib (AZD5363)	*AKT*	investigational drug in clinical trials for various types of cancer, including breast cancer, prostate cancer, and non-small cell lung cancer	[65]
Miransertib (ARQ092)	*AKT*	investigational drug in clinical trials for various types of cancer, including endometrial cancer, solid tumors, and proteus syndrome	[66]
Afuresertib (GSK2110183)	*AKT*	investigational drug in clinical trials for multiple myeloma and other hematologic malignancies	[67]
Uprosertib (GSK2141795)	*AKT*	investigational drug in clinical trials for various types of cancer, including solid tumors and lymphomas	[68]
MK-2206	*AKT*	investigational drug in clinical trials for various types of cancer, including breast cancer, colorectal cancer, and non-small cell lung cancer	[69]
Everolimus (Afinitor, Zortress)	*mTOR*	advanced renal cell carcinoma (RCC), progressive neuroendocrine tumors of pancreatic origin (PNET), advanced hormone receptor-positive, HER2-negative breast cancer, renal angiomyolipoma with tuberous sclerosis complex (TSC), and subependymal giant cell astrocytoma (SEGA) associated with TSC	[70,71]
Temsirolimus (Torisel)	*mTOR*	advanced renal cell carcinoma (RCC)	[72]
Sirolimus (Rapamune)	*mTOR*	potential anti-cancer properties in certain cancers, such as TSC-associated lymphangioleiomyomatosis (LAM)	[73]
Ridaforolimus (AP23573, MK-8669)	*mTOR*	investigational drug in clinical trials for various types of cancer, including sarcomas, endometrial cancer, and other solid tumors	[74]
Sapanisertib (INK128, TAK-228)	*mTOR*	investigational drug in clinical trials for various types of cancer, including breast cancer, renal cell carcinoma, and non-Hodgkin’s lymphoma	[75]
AZD2014 (Vistusertib)	*mTOR*	investigational drug in clinical trials for various types of cancer, including endometrial cancer, breast cancer, and non-small cell lung cancer	[76]
Dactolisib (BEZ235)	dual PI3K/*mTOR*	preclinical and early-phase clinical trials for various types of solid tumors, including breast, prostate, and renal cell carcinoma	[77]
Apitolisib (GDC-0980)	dual PI3K/*mTOR*	early-phase clinical trials for various types of solid tumors, including colorectal, breast, and prostate cancer	[78,79]
Bimiralisib (PQR309)	dual PI3K/*mTOR*	early-phase clinical trials for various types of solid tumors and lymphomas	[80]
Omipalisib (GSK2126458)	dual PI3K/*mTOR*	early-phase clinical trials for various types of solid tumors and hematologic malignancies	[62,81]
Gedatolisib (PF-05212384)	dual PI3K/*mTOR*	early-phase clinical trials for various types of solid tumors and hematologic malignancies	[82]
Vistusertib (AZD2014)	dual PI3K/*mTOR*	early-phase clinical trials for various types of solid tumors and hematologic malignancies	[83]
Serabelisib (INK1117, MLN0128, TAK-228)	dual PI3K/*mTOR*	early-phase clinical trials for various types of solid tumors and hematologic malignancies	[84,85]

**Table 3:** Functional roles of *FOXO* transcription factors in cell Biology and cancer.

*FOXO* Type	Role in Cell Biology	Role in Cancer	Types of Tumors	References
*FOXO*1	Regulation of gluconeogenesis, cell proliferation, apoptosis, metabolism, inflammation, differentiation, and stress resistance. Global deletion causes embryonic cell death due to incomplete vascular development.	Tumor suppressor, regulation of cell cycle arrest, apoptosis, and DNA repair	Lymphoma, soft tissue sarcoma, acute myeloid leukemia (AML), breast cancer	[90]
*FOXO*2	Involved in multiple important biological processes, such as cell cycle arrest, DNA repair, apoptosis, glucose metabolism, aging, and autophagy.	Tumor suppressor, regulation of cell cycle arrest, apoptosis, and DNA repair	Not specified	[90]
*FOXO*3	Affects lymph proliferation, widespread organ inflammation. Expression found in most tissues, including lymphocytes and myeloid cells.	Tumor suppressor, regulation of cell cycle arrest, apoptosis, and DNA repair	Neuroblastoma, breast cancer, colorectal cancer, glioblastoma, pancreatic ductal adenocarcinoma	[90]
*FOXO*4	Required for stem cell function in multiple tissues, including the maintenance of hematopoietic, neural, and muscle stem cell pools.	Tumor suppressor, regulation of cell cycle arrest and apoptosis	Not specified	[90]
